# Thermodynamic Performance of a Brayton Pumped Heat Energy Storage System: Influence of Internal and External Irreversibilities

**DOI:** 10.3390/e23121564

**Published:** 2021-11-24

**Authors:** David Pérez-Gallego, Julian Gonzalez-Ayala, Antonio Calvo Hernández, Alejandro Medina

**Affiliations:** 1 Departamento de Física Aplicada, Universidad de Salamanca, 37008 Salamanca, Spain; id00698231@usal.es (D.P.-G.); anca@usal.es (A.C.H.); amd385@usal.es (A.M.); 2 Instituto Universitario de Física Fundamental y Matemáticas (IUFFyM), Universidad de Salamanca, 37008 Salamanca, Spain

**Keywords:** energy storage, round-trip efficiency, irreversibilities, Brayton cycle, heat pump

## Abstract

A model for a pumped thermal energy storage system is presented. It is based on a Brayton cycle working successively as a heat pump and a heat engine. All the main irreversibility sources expected in real plants are considered: external losses arising from the heat transfer between the working fluid and the thermal reservoirs, internal losses coming from pressure decays, and losses in the turbomachinery. Temperatures considered for the numerical analysis are adequate for solid thermal reservoirs, such as a packed bed. Special emphasis is paid to the combination of parameters and variables that lead to physically acceptable configurations. Maximum values of efficiencies, including round-trip efficiency, are obtained and analyzed, and optimal design intervals are provided. Round-trip efficiencies of around 0.4, or even larger, are predicted. The analysis indicates that the physical region, where the coupled system can operate, strongly depends on the irreversibility parameters. In this way, maximum values of power output, efficiency, round-trip efficiency, and pumped heat might lay outside the physical region. In that case, the upper values are considered. The sensitivity analysis of these maxima shows that changes in the expander/turbine and the efficiencies of the compressors affect the most with respect to a selected design point. In the case of the expander, these drops are mostly due to a decrease in the area of the physical operation region.

## 1. Introduction

Increasing production of electric energy from renewable sources like wind or Sun makes essential energy storage technologies due to the inherent intermittency of such natural resources [1]. Substantial efforts are being devoted to the so-called Pumped Thermal Energy Storage (PTES) systems [2]. They are aimed at storing energy during the hours with an excess of, for instance, wind or photovoltaic production. When electric energy is subsequently required, heat is transformed again in electricity through some thermodynamic cycle. Required storage periods could be not very long, from four to eight hours. At grid-scale, the only proven competitors of this concept are Pumped Hydro Energy Storage and Compressed Air Energy Storage [3]. Nevertheless, these technologies require very particular geographical and geological scenarios that make difficult their implementation where required [4]. On the other hand, electric batteries, as with lithium ones, are still very expensive at grid-scale.

Recently, Dumont et al. [5] have proposed the term *Carnot batteries* to those systems that are used to store energy by establishing a temperature difference between two media (low and high temperature reservoirs). In the charge period, energy is stored (usually by means of a thermal heat pump). And after a storage period, electric energy is produced by a heat engine that works between those reservoirs. Several configurations and thermodynamic cycles can be considered [6]. To date, only a few prototype installations were built [7], and it is still necessary a research work at a fundamental level to provide optimal plant configurations and adequate intervals for the most significant design parameters. This work is focused on Brayton-like cycles [8] and constant temperature heat reservoirs as packed bed solid reservoirs at equilibrium.

The working fluids in Brayton-like thermodynamic cycles are gases stable at high temperatures, chemically inert, cheap, and environmentally friendly [9]. These requirements limit the options to Ar and N${}_{2}$ (or air). Ar is actually the preferred option, because it can reach higher temperatures for the same storage pressure ratios [5], although there is more experimental background in the turbomachinery working with air. In any case, the ideal gas equation of state is sufficient for making efficiency and cost estimations. Moreover, in the case of Ar, specific heats are temperature independent. Packed bed thermal storage systems have been widely analyzed in the literature. A recent review on types of packed bed systems, the most relevant experimental works in the last years, and their numerical modeling are due to Esence et al. [10].

The use of packed bed sensible heat storage makes it possible to reach high temperatures, and there is a direct heat transfer between the working fluid in the thermodynamic cycle and the reservoir itself [5]. This avoids the use of intermediate heat exchangers, and overall efficiency is supposedly higher. Moreover, only one deposit at a high temperature is required at difference with the case of liquid storage. This kind of thermal storage is especially interesting for not overly long storage periods [5], for instance, of the order of several hours, and for Brayton cycles, whose efficiency when working as a heat engine is large for high turbine inlet temperatures.

When modeling this type of PTES configuration, several key points should be taken into account: the losses in the heat transfer between the gas working fluid and the solid particulates, the pressure decay in the heat exchanges, the efficiencies of expanders and compressors, and the heat leakage through the containers walls during storage. It has been demonstrated that in optimized designs, the round-trip efficiency is more sensitive to the performance of expanders, turbines, and compressors than to the losses coming from the packed bed thermal storage towards the surroundings [5]. As stated by McTigue et al. [11] heat leakage can be reduced to any desired level with an appropriate investment in storage insulation. Numerically a 1% heat leakage from each reservoir could be reflected in a reduction of about 2% in round-trip efficiency.

The objective of the present study is to propose a thorough thermodynamic model for the overall round-trip performance of a PTES system composed of a Brayton heat pump and a Brayton heat engine during charge and discharge periods, respectively, and considering solid storage reservoirs at an approximately constant temperature. All internal and external irreversibilities in heat exchanges and during compression and expansion processes will be considered. The system will be assumed to be at stationary conditions with fixed temperature reservoirs to obtain analytical equations allowing for detailed sensitivity analyses of the influence of all possible losses and different temperature levels in system performance. In previous studies by our group [12,13], PTES Brayton-like systems with liquid storage and their optimization were developed. This work is intended to present a complete and flexible thermodynamic model capable to include all the most relevant peculiarities and thermodynamic details emerging from the combination of Brayton-like heat devices and solid thermal reservoirs. Special emphasis is placed on the links between charge and discharge cycles, pressure drops during heat exchanges, and the losses in turbines, expanders, and compressors. The relative importance of external and internal irreversibilities is analyzed, as well as the influence of each of them in the optimal values of objective functions, such as round-trip efficiency or power output.

## 2. Thermodynamic Model

The PTES storage system is made up of two irreversible Brayton engines, one working as a heat pump and another as a heat engine. The first operates in a charge stage, from which heat is transferred to the hot reservoir and energy is stored. This is used later as an input heat for the discharge stage performed by the heat engine. The working fluid interacts with two reservoirs (hot or cold storage) at a constant temperature. In this process, energy is used or produced. The combination in a sequence of both cycles with a time delay between them constitutes the energy storage system. That time delay is equivalent to the intended storage period.

In the model developed it will be considered that heat reservoirs operate at a constant temperature, i.e., after a non-stationary transient period they reach an equilibrium temperature. This simplified stationary model will allow for detailed sensitivity analysis of the key thermodynamic parameters of the system and to optimize the elected objective functions. Thus, thermodynamic optimum pre-design intervals for the most influential parameters will be proposed. Another key hypothesis is that working fluid specific heats are approximately constant in the temperature interval considered. This is true for Ar or other noble gases and is not a bad supposition for diatomic gases such as air. In this way, the heat capacity of the working fluid per unit time, $C_{w}$, that depends on the mass flow, $\dot{m}$, and the specific heat at constant pressure, $c_{p,w}$, can be written as: $C_{w}=\dot{m}c_{p,w}$.

### 2.1. Thermodynamic Analysis: Heat Pump Cycle

**Figure 1:**
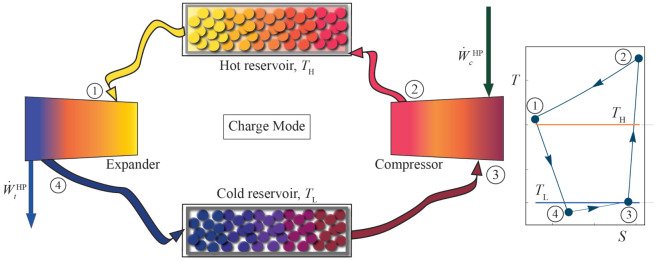
Components scheme of the Brayton-like HP charge cycle and the approximate T−S diagram in the inset at the right. The processes 2→1 and 4→3 have been represented as isobaric (ideal), therefore they do not end at the point corresponding to the final state, because of the pressure drops. Hereinafter, realistic processes including losses will be shown.

A complete (round-trip) cycle begins with the charge cycle. It is considered as an inverse Brayton thermodynamic cycle. The working fluid absorbs heat from a cold storage at temperature $T_{L}$, and using a network from the external energy source (wind, PV, or whichever) through a compressor, it is compressed by increasing its pressure and temperature. In this way, it can transfer heat to a hot storage at $T_{H}$. In the process, energy is then released in an expander, and gas temperature and pressure decrease in an approximately isentropic path. A components scheme of this cycle is shown in Figure 1.

The charge cycle, in a T−S diagram, is shown in the inset of Figure 1. It runs in an anti-clockwise direction. The compressor increases working fluid pressure (and temperature) from state 3 to state 2 (3→2). Ideally, it is a reversible isentropic compression, but the model considers an entropy increase through the consideration of isentropic compressor efficiency, $\epsilon_{c}^{HP}\leq 1$, defined as:

(1) $$\epsilon_{c}^{HP}=\frac{{\dot{W}}_{c,\;ideal}^{HP}}{{\dot{W}}_{c}^{HP}}=\frac{T_{2S}-T_{3}}{T_{2}-T_{3}},$$

where $T_{2S}$ represents the temperature the working fluid eventually would reach after an ideal compression. Thus, $\epsilon_{c}^{HP}$, is the ratio between the work required in ideal conditions and the one consumed in real conditions.

At state 2, the working fluid reaches the maximum pressure and temperature in the cycle. Then, the working fluid releases heat to the hot storage (process 2→1). This process is isobaric in the ideal case. To account for the real pressure losses, $\Delta p_{H}$, a pressure decay parameter is defined [12]:

(2) $$\rho_{H}^{HP}={\left(\frac{p_{1}}{p_{2}}\right)}^{\kappa }={\left(\frac{p_{2}-\Delta p_{H}}{p_{2}}\right)}^{\kappa },$$

where κ≡(γ−1)/γ is defined by convenience and γ is the adiabatic coefficient of the working fluid that is considered approximately constant in the working temperature intervals.

At state 1, the working fluid has reduced its temperature, due to the transfer of heat to the hot storage. Subsequently, the working fluid enters the expander. Its temperature and pressure decrease up to state 4 (1→4). Like in compression, the isentropic expander efficiency is considered to account for losses:

(3) $$\epsilon_{t}^{HP}=\frac{{\dot{W}}_{t}^{HP}}{{\dot{W}}_{t,\;ideal}^{HP}}=\frac{T_{1}-T_{4}}{T_{1}-T_{4S}}.$$

Finally, and to close the thermodynamic cycle, the fluid absorbs heat from a cold storage. This storage is at a lower temperature than the hot storage, but at a higher temperature than the working fluid in state 4. The temperature of the cold reservoir is denoted as $T_{L}$. The heat absorption in step 4→3 ideally is an isobaric process, but pressure drops in the real process are quantified through:

(4) $$\rho_{L}^{HP}={\left(\frac{p_{3}}{p_{4}}\right)}^{\kappa }={\left(\frac{p_{4}-\Delta p_{L}}{p_{4}}\right)}^{\kappa },$$

For the correct operation of the charge cycle, the temperatures of each state must comply in a sequential order. The temperature of each state is limited as follows.

(5) $$0\leq T_{4}^{HP}\leq T_{3}^{HP}\leq T_{L}\leq T_{H}\leq T_{1}^{HP}\leq T_{2}^{HP}.$$

The work per unit time consumed by the compressor and the work per unit of time released at the expander are:

(6) $$\begin{array}{cc} {\dot{W}}_{c}^{HP}=C_{w}\left(T_{2}-T_{3}\right); & {\dot{W}}_{t}^{HP}=C_{w}\left(T_{1}-T_{4}\right), \end{array}$$

so, the power output required to run the pump can be written as:

(7) $$P_{in}={\dot{W}}_{in}={\dot{W}}_{c}^{HP}-{\dot{W}}_{t}^{HP}=C_{w}\left(T_{2}-T_{3}-T_{1}+T_{4}\right),$$

where ${\dot{W}}_{c}^{HP}>{\dot{W}}_{t}^{HP}$, in absolute value. The heat per unit time that the working fluid transfers to the hot storage in step 2 → 1, is ${\dot{Q}}_{H}^{HP}=C_{w}\left(T_{2}-T_{1}\right)$. Similarly, the working fluid absorbs heat from a cold storage in step 4 → 3, ${\dot{Q}}_{L}^{HP}=C_{w}\left(T_{3}-T_{4}\right)$. The pressure ratios of the compressor and expander can be expressed as: $r_{c}^{HP}=p_{2}/p_{3}$ and $r_{t}^{HP}=p_{1}/p_{4}$. These parameters give us the relationship between the pressure of the working fluid in the inlet and the outlet of the compressor and expander. In terms of the temperatures it is easy to show that:

(8) $$a_{c}^{HP}=\frac{T_{2S}}{T_{3}}=r_{c}^{\kappa }={\left(\frac{p_{2}}{p_{3}}\right)}^{\kappa }={\left(\frac{p_{2}}{p_{4}-\Delta p_{L}}\right)}^{\kappa },$$

(9) $$a_{t}^{HP}=\frac{T_{1}}{T_{4S}}=r_{t}^{\kappa }={\left(\frac{p_{1}}{p_{4}}\right)}^{\kappa }={\left(\frac{p_{2}-\Delta p_{H}}{p_{4}}\right)}^{\kappa },$$

and so, there is a connection among these parameters:

(10) $$a_{t}^{HP}=a_{c}^{HP}\rho_{H}^{HP}\rho_{L}^{HP}.$$

The new parameters were introduced to allow us to rewrite the work consumed by the compressor and the work done by the turbine. Using Equations (1), (3), (8) and (9):

(11) $${\dot{W}}_{c}^{HP}=C_{w}\left(T_{2}-T_{3}\right)=C_{w}T_{3}\left(\frac{a_{c}-1}{\epsilon_{c}}\right),$$

(12) $${\dot{W}}_{t}^{HP}=C_{w}\left(T_{1}-T_{4}\right)=C_{w}T_{1}\epsilon_{t}\left(\frac{a_{t}-1}{a_{t}}\right).$$

It is considered that there are losses in the heat transfers between the working fluid and the reservoirs. To quantify these external irreversibilities, the maximum heats which the working fluid would exchange under ideal conditions, reaching the temperatures of the hot storage ($T_{H}$) and the cold storage ($T_{L}$) would be:

(13) $${\dot{Q}}_{H,max}^{HP}=C_{w}\left(T_{2}-T_{H}\right),$$

(14) $${\dot{Q}}_{L,max}^{HP}=C_{w}\left(T_{L}-T_{4}\right).$$

The parameters which characterize these external irreversibilities can be defined as the ratios between the actual heat transferred and the maximum heat transferred under ideal conditions.

(15) $$\epsilon_{H}^{HP}=\frac{{\dot{Q}}_{H}^{HP}}{{\dot{Q}}_{H,max}^{HP}}=\frac{T_{2}-T_{1}}{T_{2}-T_{H}},$$

(16) $$\epsilon_{L}^{HP}=\frac{{\dot{Q}}_{L}^{HP}}{{\dot{Q}}_{L,max}^{HP}}=\frac{T_{3}-T_{4}}{T_{L}-T_{4}}.$$

In a heat pump, the coefficient of performance (COP), ν, quantifies the quotient between the heat which the fluid releases to the hot storage and the work input used for that task:

(17) $$\nu \equiv \frac{{\dot{Q}}_{H}^{HP}}{{\dot{W}}_{in}}=\frac{{\dot{Q}}_{H}^{HP}}{{\dot{Q}}_{H}^{HP}-{\dot{Q}}_{L}^{HP}}=\frac{T_{2}-T_{1}}{T_{2}-T_{1}-T_{3}+T_{4}}.$$

In the last equality, it was used that the internal energy variation in a cyclic process is zero. All of the temperatures and, therefore, the COP can be written analytically in terms of geometric parameters (pressure and temperature ratios, and the adiabatic coefficient of the working fluid) and the parameters defined to account for both internal ( $\rho_{H}^{HP}$, $\rho_{L}^{HP}$, $\epsilon_{c}^{HP}$, $\epsilon_{t}^{HP}$) and external irreversibilities ($\epsilon_{H}^{HP}$,$\epsilon_{L}^{HP}$). From Equations (1), (3), (10), (15) and (16) the four temperatures and $a_{t}^{HP}$ can be expressed in terms of the irreversible parameters and $a_{c}^{HP}$, resulting in:

(18) $$T_{1}=-\frac{a_{c}^{HP}\rho_{H}^{HP}\rho_{L}^{HP}\left[T_{L}\left(\epsilon_{H}^{HP}-1\right)\epsilon_{L}^{HP}\left(a_{c}^{HP}+\epsilon_{c}^{HP}-1\right)T_{H}\epsilon_{c}^{HP}\epsilon_{H}^{HP}\right]}{D^{HP}},$$

where $D^{HP}$ is use to simplify the expression, being

(19) $$\begin{array}{cc} D^{HP}= & \epsilon_{t}^{HP}\left(\epsilon_{H}^{HP}-1\right)\left(\epsilon_{L}^{HP}-1\right)\left(a_{c}^{HP}+\epsilon_{c}^{HP}-1\right)\left(a_{c}^{HP}\rho_{H}^{HP}\rho_{L}^{HP}-1\right)-\\ \; & a_{c}^{HP}\rho_{H}^{HP}\rho_{L}^{HP}\left[\epsilon_{L}^{HP}\left(\epsilon_{H}^{HP}-1\right)\left(a_{c}^{HP}+\epsilon_{c}^{HP}-1\right)-a_{c}^{HP}\epsilon_{H}^{HP}+a_{c}^{HP}-\epsilon_{c}^{HP}\epsilon_{H}^{HP}+\epsilon_{H}^{HP}-1\right], \end{array}$$

in the same way, the other temperatures are

(20) $$T_{2}=\frac{\left(a_{c}^{HP}+\epsilon_{c}^{HP}-1\right)\left\{T_{H}\epsilon_{H}^{HP}\left(\epsilon_{L}^{HP}-1\right)\left[a_{c}^{HP}\rho_{H}^{HP}\rho_{L}^{HP}\left(\epsilon_{t}^{HP}-1\right)-\epsilon_{t}^{HP}\right]+a_{c}^{HP}\rho_{H}^{HP}\rho_{L}^{HP}T_{L}\epsilon_{L}^{HP}\right\}}{D^{HP}},$$

(21) $$T_{3}=\frac{\epsilon_{c}^{HP}\left\{T_{H}\epsilon_{H}^{HP}\left(\epsilon_{L}^{HP}-1\right)\left[a_{c}^{HP}\rho_{H}^{HP}\rho_{L}^{HP}\left(\epsilon_{t}^{HP}-1\right)-\epsilon_{t}^{HP}\right]+a_{c}^{HP}\rho_{H}^{HP}\rho_{L}^{HP}T_{L}\epsilon_{L}^{HP}\right\}}{D^{HP}},$$

(22) $$T_{4}=-\frac{\left[a_{c}^{HP}\rho_{H}^{HP}\rho_{L}^{HP}\left(\epsilon_{t}^{HP}-1\right)-\epsilon_{t}^{HP}\right]\left[T_{H}\epsilon_{c}^{HP}\epsilon_{H}^{HP}-T_{L}\left(\epsilon_{H}^{HP}-1\right)\epsilon_{L}^{HP}\left(a_{c}^{HP}+\epsilon_{c}^{HP}-1\right)\right]}{a_{c}^{HP}\rho_{H}^{HP}\rho_{L}^{HP}\epsilon_{c}^{HP}+\left(\epsilon_{H}^{HP}-1\right)\left(\epsilon_{L}^{HP}-1\right)\left(a_{c}^{HP}+\epsilon_{c}^{HP}-1\right)\left[a_{c}^{HP}\rho_{H}^{HP}\rho_{L}^{HP}\left(\epsilon_{t}^{HP}-1\right)-\epsilon_{t}^{HP}\right]},$$

### 2.2. Thermodynamic Analysis: Heat Engine Cycle

**Figure 2:**
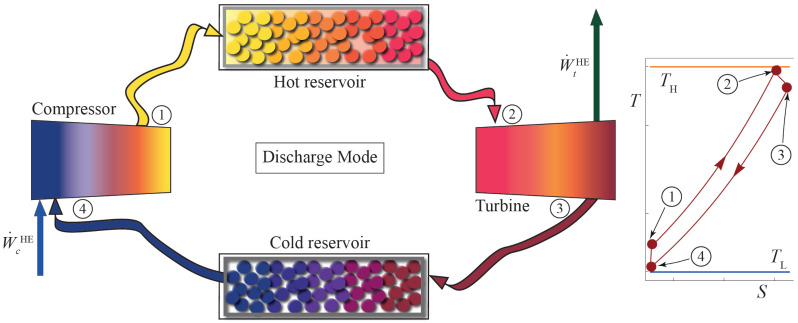
Scheme of the discharge Brayton cycle and its corresponding T−S diagram in the inset.

The discharge cycle after the storage period is also considered as a Brayton-like thermodynamic cycle, in this case corresponding to a heat engine. This cycle is analogous to the charge one, but the working fluid runs in the opposite direction in the T−S diagram, in a clockwise direction (see Figure 2).

In the discharge cycle, the working fluid enters the compressor at state 4. Working fluid is compressed up to state 1 (4→1). Losses in the compressor are accounted for through its isentropic efficiency, $\epsilon_{c}^{HE}$. Afterward, in the heat input process (1→2) the working fluid is heated up to the turbine inlet temperature. Pressure decay in the heat input is considered through the parameter $\rho_{H}^{HE}$. Irreversibilities in the heat transfer from the reservoir to the working fluid are quantified with the parameter $\epsilon_{H}^{HE}$. Subsequently, the working fluid enters the turbine at its maximum temperature, $T_{2}$. The expansion to state 3 (2→3) is also considered as non-isentropic. This internal irreversibility is considered through $\epsilon_{t}^{HE}$. Finally, a heat release to the cold storage allows for a cyclic process for the working fluid. The corresponding external irreversibilities are represented by $\epsilon_{H}^{HE}$. For the correct operation of the discharge cycle, the temperatures of each state comply with an order analogous to the charge cycle. The temperature of each state is limited as follows:

(23) $$T_{L}\leq T_{4}^{HE}\leq T_{3}^{HE}\leq T_{2}^{HE}\leq T_{H},$$

(24) $$T_{L}\leq T_{4}^{HE}\leq T_{1}^{HE}\leq T_{2}^{HE}\leq T_{H}.$$

Internal and external irreversibilities parameters are defined next:

(25) $$\begin{array}{cc} \epsilon_{c}^{HE}=\frac{{\dot{W}}_{c,\;ideal}^{HE}}{{\dot{W}}_{c}^{HE}}=\frac{T_{1S}-T_{4}}{T_{1}-T_{4}}; & \epsilon_{t}^{HE}=\frac{{\dot{W}}_{t}^{HE}}{{\dot{W}}_{t,\;ideal}^{HE}}=\frac{T_{2}-T_{3}}{T_{2}-T_{3S}} \end{array}$$

(26) $$\begin{array}{cc} \rho_{H}^{HE}={\left(\frac{p_{2}}{p_{1}}\right)}^{\kappa }={\left(\frac{p_{1}-\Delta p_{H}}{p_{1}}\right)}^{\kappa }; & \rho_{L}^{HE}={\left(\frac{p_{4}}{p_{3}}\right)}^{\kappa }={\left(\frac{p_{3}-\Delta p_{L}}{p_{3}}\right)}^{\kappa } \end{array}$$

(27) $$\begin{array}{cc} \epsilon_{H}^{HE}=\frac{{\dot{Q}}_{H}^{HE}}{{\dot{Q}}_{H,max}^{HE}}=\frac{T_{2}-T_{1}}{T_{H}-T_{1}}; & \epsilon_{L}^{HE}=\frac{{\dot{Q}}_{L}^{HE}}{{\dot{Q}}_{L,max}^{HE}}=\frac{T_{3}-T_{4}}{T_{3}-T_{L}}. \end{array}$$

The work per unit time required by the compressor and the one produced by the turbine are:

(28) $$\begin{array}{cc} {\dot{W}}_{c}^{HE}=C_{w}\left(T_{1}-T_{4}\right); & {\dot{W}}_{t}^{HE}=C_{w}\left(T_{2}-T_{3}\right), \end{array}$$

so, the power output is:

(29) $$P_{out}={\dot{W}}_{out}={\dot{W}}_{c}^{HE}-{\dot{W}}_{t}^{HE}=C_{w}\left(T_{1}-T_{4}-T_{2}+T_{3}\right),$$

The heat per unit time the working fluid absorbs from the hot storage in step 1 → 2, is defined as follows:

(30) $${\dot{Q}}_{H}^{HE}=C_{w}\left(T_{2}-T_{1}\right).$$

The temperature of the working fluid at state 2 must be lower than the temperature of the hot storage because the heat is transferred from the storage to the working fluid. In the same way, the working fluid releases heat to a cold storage in step 3 → 4. The temperature of the working fluid in state 4 must be higher than the temperature of the cold reservoir.

(31) $${\dot{Q}}_{L}^{HE}=C_{w}\left(T_{3}-T_{4}\right).$$

The pressure ratios of the compressor and the turbine are:

(32) $$\begin{array}{cc} r_{c}^{HE}=\frac{p_{1}}{p_{4}}; & r_{t}^{HE}=\frac{p_{2}}{p_{3}}, \end{array}$$

and the temperature ratios,

(33) $$\begin{array}{cc} a_{c}^{HE}=\frac{T_{1S}}{T_{4}}=r_{c}^{\kappa }; & a_{t}^{HE}=\frac{T_{2}}{T_{3S}}=r_{t}^{\kappa }, \end{array}$$

so,

(34) $$a_{t}^{HE}=a_{c}^{HE}\rho_{H}^{HE}\rho_{L}^{HE}.$$

The new parameters introduced allow us to rewrite the work consumed by the compressor and the work done by the turbine as:

(35) $$\begin{array}{cc} {\dot{W}}_{c}^{HE}=C_{w}T_{4}\left(\frac{a_{c}-1}{\epsilon_{c}}\right); & {\dot{W}}_{t}^{HE}=C_{w}T_{2}\;\epsilon_{t}\left(\frac{a_{t}-1}{a_{t}}\right). \end{array}$$

Heat engine efficiency (η) quantifies the amount of useful energy in relation to the amount of heat input from the hot storage

(36) $$\eta \equiv \frac{{\dot{W}}_{out}}{{\dot{Q}}_{H}^{HE}}=\frac{P_{out}^{HE}}{{\dot{Q}}_{H}^{HE}}=\frac{T_{1}-T_{4}-T_{2}+T_{3}}{T_{2}-T_{1}}.$$

All the cycle temperatures and therefore its efficiency can be expressed in terms of pressure and temperature ratios, and the parameters defined to account for internal (pressure losses and non-ideality of compressor and turbine) and external irreversibilities (heat transfer from the reservoirs to the working fluid). The following analytical expressions for cycle temperature are found from Equations (25), (27) and (34), in this case:

(37) $$T_{1}=\frac{\left(a_{c}^{HE}+\epsilon_{c}^{HE}-1\right)\left\{T_{H}\epsilon_{H}^{HE}\left(\epsilon_{L}^{HE}-1\right)\left[a_{c}^{HE}\rho_{H}^{HE}\rho_{L}^{HE}\left(\epsilon_{t}^{HE}-1\right)-\epsilon_{t}^{HE}\right]+a_{c}^{HE}\rho_{H}^{HE}\rho_{L}^{HE}T_{L}\epsilon_{L}^{HE}\right\}}{D^{HE}}$$

where, once again, the term $D^{HE}$ is introduced to simplify the expression, being

(38) $$\begin{array}{cc} D^{HE}= & \epsilon_{t}^{HE}\left(\epsilon_{H}^{HE}-1\right)\left(\epsilon_{L}^{HE}-1\right)\left(a_{c}^{HE}+\epsilon_{c}^{HE}-1\right)\left(a_{c}^{HE}\rho_{H}^{HE}\rho_{L}^{HE}-1\right)-\\ \; & a_{c}^{HE}\rho_{H}^{HE}\rho_{L}^{HE}\left[\epsilon_{L}^{HE}\left(\epsilon_{H}^{HE}-1\right)\left(a_{c}^{HE}+\epsilon_{c}^{HE}-1\right)-a_{c}^{HE}\epsilon_{H}^{HE}+a_{c}^{HE}-\epsilon_{c}^{HE}\epsilon_{H}^{HE}+\epsilon_{H}^{HE}-1\right], \end{array}$$

in the same way, the other temperatures are

(39) $$T_{2}=\frac{a_{c}^{HE}\rho_{H}^{HE}\rho_{L}^{HE}\left[T_{H}\epsilon_{c}^{HE}\epsilon_{H}^{HE}-T_{L}\left(\epsilon_{H}^{HE}-1\right)\epsilon_{L}^{HE}\left(a_{c}^{HE}+\epsilon_{c}^{HE}-1\right)\right]}{D^{HE}},$$

(40) $$T_{3}=-\frac{\left[a_{c}^{HE}\rho_{H}^{HE}\rho_{L}^{HE}\left(\epsilon_{t}^{HE}-1\right)-\epsilon_{t}^{HE}\right]\left[T_{H}\epsilon_{c}^{HE}\epsilon_{H}^{HE}-T_{L}\left(\epsilon_{H}^{HE}-1\right)\epsilon_{L}^{HE}\left(a_{c}^{HE}+\epsilon_{c}^{HE}-1\right)\right]}{D^{HE}},$$

(41) $$T_{4}=-\frac{\epsilon_{c}^{HE}\left\{-T_{H}\epsilon_{H}^{HE}\left(\epsilon_{L}^{HE}-1\right)\left[a_{c}^{HE}\rho_{H}^{HE}\rho_{L}^{HE}\left(\epsilon_{t}^{HE}-1\right)-\epsilon_{t}^{HE}\right]-a_{c}^{HE}\rho_{H}^{HE}\rho_{L}^{HE}T_{L}\epsilon_{L}^{HE}\right\}}{a_{c}^{HE}\rho_{H}^{HE}\rho_{L}^{HE}\epsilon_{c}^{HE}+\left(\epsilon_{H}^{HE}-1\right)\left(\epsilon_{L}^{HE}-1\right)\left(a_{c}^{HE}+\epsilon_{c}^{HE}-1\right)\left[a_{c}^{HE}\rho_{H}^{HE}\rho_{L}^{HE}\left(\epsilon_{t}^{HE}-1\right)-\epsilon_{t}^{HE}\right]},$$

### 2.3. Round-Trip Efficiency

**Figure 3:**
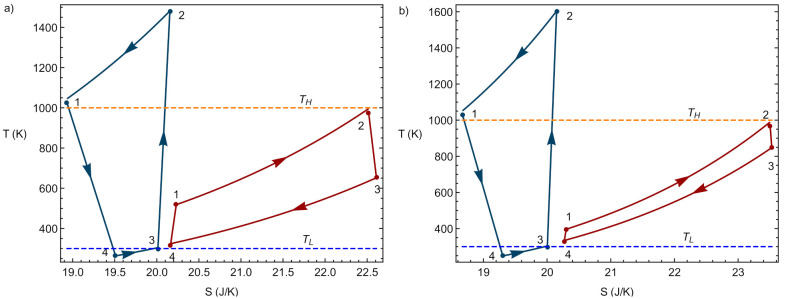
Two illustrative examples of the coupling of charge and discharge cycles in T−S diagrams. Values considered for the temperatures of the reservoirs: $T_{H}=1000$ K, $T_{L}=300$ K. In (**a**) the case where $a_{c}^{HE}$ = 1.8 and $a_{c}^{HP}$ = 4.57, and in (**b**) the case $a_{c}^{HE}$ = 1.2 and $a_{c}^{HP}$ c = 5.17.

Once the charge and discharge phases of the system have been modeled separately, a global or round-trip cycle can be defined and analyzed. The global cycle starts with the charging of heat towards the hot storage. The energy is stored for the required period of time, and later on it is converted into electric energy again through the discharge heat engine cycle. Each thermodynamic charge or discharge phase can be carried out one or multiple times depending on the energy demand and the dimensions of the PTES system. In this work, the PTES system will be analyzed over a single charge and discharge sequence. Figure 3 contains two representative examples of the coupling of charge and discharge cycles.

Overall performance of the energy storage cycle is usually evaluated through the round-trip efficiency, Φ [14]. It is defined as the ratio between the net energy obtained from storage during discharge and the net energy introduced into the system during the charge.

(42) $$\Phi =\frac{W_{out}}{W_{in}}=\frac{P_{out}}{P_{in}},$$

where $W_{out}$ refers to the useful energy obtained from storage, $W_{in}$ is the work input in the charge stage. $P_{out}$ and $P_{in}$ are the electrical net powers from the discharge and charge phases. For the last equality identical charge and discharge times are assumed in this model. Recently, the analysis of the case in which the charge and discharge times are different has been studied in an endoreversible machine model, studying the time influence on the performances for different operating regimes [15].

The performance of heat devices is obviously limited by the maximum theoretical efficiency of Carnot. This efficiency depends on the temperature of the storages between which the cycle operates. The individual charge and discharge efficiencies are linked to this theoretical restriction. However, the round-trip efficiency quantifies the energy obtained from the stored energy. In this process, the heat used in the discharge phase comes from the energy stored in the charge phase, $Q_{H}^{HP}$. The round-trip efficiency of the overall cycle, Φ is not limited by the theoretical Carnot efficiency. Using the definitions of COP in the charging mode and efficiency in the discharge phase the round-trip efficiency can be written as,

(43) $$\Phi =\eta \nu \frac{{\dot{Q}}_{H}^{HE}}{{\dot{Q}}_{H}^{HP}}.$$

If the energy stored in the hot storage is the same that the heat used for the discharge phase (without heat leak storage losses) then ${\dot{Q}}_{H}^{HP}={\dot{Q}}_{H}^{HE}$ and in consequence the round-trip efficiency is:

(44) Φ=ην.

The condition ${\dot{Q}}_{H}^{HP}={\dot{Q}}_{H}^{HE}$ constitutes an idealization in this analysis, providing upper values for the coupled system performance. This constraint establishes a relationship between the compression ratios of the working fluid at the inlet and outlet of the compressor in the charge/discharge phases, from which $a_{c}^{HP}=a_{c}^{HP}\left(a_{c}^{HE}\right)$.

### 2.4. Endoreversible Limit

**Figure 4:**
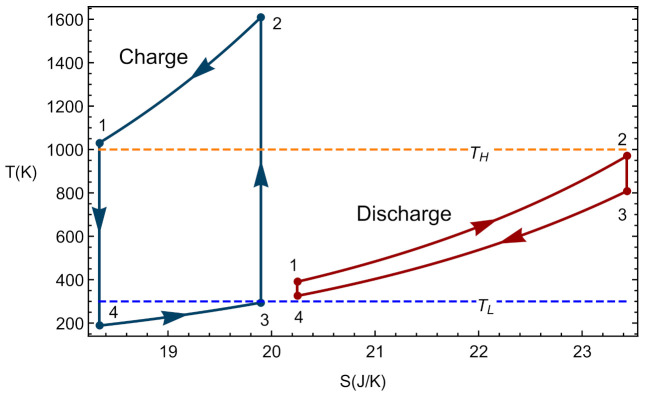
Example of an endoreversible coupled cycle. The figures correspond to the following parameters: $a_{c}^{HE}=1.2$, $a_{c}^{HP}=5.47$, $\epsilon_{c}^{HP,\;HE}=\epsilon_{t}^{HP,\;HE}=1$, $\rho_{H}^{HP,\;HE}=\rho_{L}^{HP,\;HE}=1$, $\epsilon_{H}^{HP,\;HE}=\epsilon_{L}^{HP,\;HE}=0.95$, $T_{H}=1000$ K, and $T_{L}=300$ K.

In the framework of the model considered, it is possible to recover the endoreversible limit as a particular case where the only losses come from the external irreversibilities due to the coupling of the working fluid with the heat storage, i.e., in this limit compressors, turbines, and expanders isentropic efficiencies are considered as 1, as well as the parameters $\rho_{H}$ and $\rho_{L}$ for charge and discharge (no pressure decays in heat inputs or releases). Thus, the overall cycle is internally reversible, and so the compression and expansion steps occur without heat exchanges between the working fluid and the surroundings. Thus, processes 3→2 and 1→4 of the charge phase or 2→3 and 4→1 in discharge phase correspond to a constant entropy vertical line in the T−S diagram. In the same way, as there are no internal irreversibilities, the pressure drops are zero in processes 1→2 and 3→4 in charge or in the steps 2→1 and 4→ 3 in discharge. They are isobaric processes for the working fluid that is assumed to behave as an ideal gas. Figure 4 depicts the typical shape of a complete cycle in the endoreversible limit. In this example, the temperatures of the hot and cold storages are set at realistic values for solid thermal storage: $T_{H}=1000$ K and $T_{L}=300$ K.

Analytical expressions for $P_{out}$ and Φ were calculated under endoreversible conditions with the aim of comparing our results with previous ones in the literature as a validation. The following expressions are found:

(45) $$\Phi =\frac{a_{c}^{HP}\left(a_{c}^{HE}-1\right)\left(T_{H}-a_{c}^{HE}T_{L}\right)}{\left(a_{c}^{HP}-1\right)a_{c}^{HE}\left(a_{c}^{HP}T_{L}-T_{H}\right)},$$

(46) $${\bar{P}}_{out}=\frac{\left(a_{c}^{HE}-1\right)\epsilon_{H}\epsilon_{L}\left(a_{c}^{HE}T_{L}-T_{H}\right)}{a_{c}^{HE}\left(\epsilon_{H}-1\right)\epsilon_{L}-a_{c}^{HE}\epsilon_{H}}.$$

Particularly, the round-trip efficiency in conditions of maximum net output power (power at the discharge), $\Phi_{maxP_{out}}$, was obtained in two steps. First, the expression of the net output power in the discharge with respect to the parameter $a_{c}^{HE}$ is maximized. And second, the value obtained for $a_{c}^{HE}$ is substituted in Φ. The results are shown in the next equations:

(47) $$a_{c,maxP_{out}}^{HE}=\sqrt{\frac{T_{H}}{T_{L}}},$$

(48) $$max{\bar{P}}_{out}=\frac{\epsilon_{H}\epsilon_{L}\left(T_{H}+T_{L}-2\sqrt{T_{H}T_{L}}\right)}{\epsilon_{H}+\epsilon_{L}-\epsilon_{H}\epsilon_{L}},$$

(49) $$\Phi_{maxP_{out}}=\frac{2\sqrt{T_{H}}-\sqrt{T_{L}}}{2\sqrt{T_{H}}+\sqrt{T_{L}}}.$$

**Figure 5:**
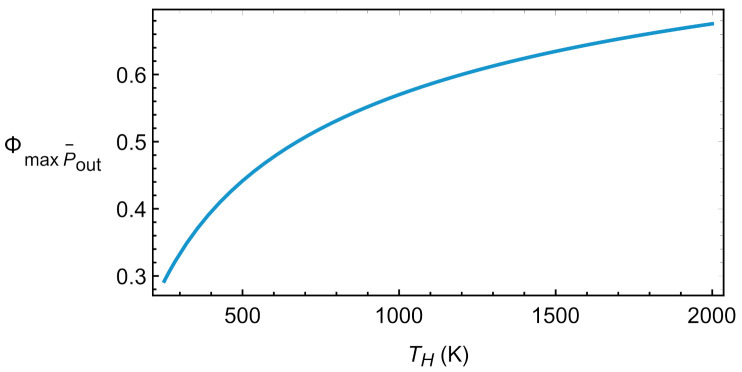
Round-trip efficiency in the endoreversible limit at maximum $P_{out}$.

These equations are the same as those provided in Refs. [15,16,17]. Although in those works the values were obtained in the context of an irreversible Carnot model, it should be noted that these types of models are equivalent to Brayton-type cycles for both engines and refrigerators, as has been demonstrated in [18,19,20] for linear transfer laws and constant heat capacities. Figure 5 depicts the evolution of $\Phi_{maxP_{out}}$ with the temperature of the hot reservoir. A monotonic increasing behavior is observed with numerical values for the round-trip efficiency relatively large, for instance about 0.57 for $T_{H}=1000$ K.

It is also possible to show that at endoreversible conditions, the value of $a_{c}$ leading to maximum round-trip efficiency is identical to that giving maximum power output, i.e., Equation (47), so maximum round-trip efficiency and power output at maximum round-trip efficiency are the same as those obtained at maximum power output, Equations (48) and (49) respectively. In summary, the proposed thermodynamic model, at the endoreversible limit, recovers previous results in the literature ensuring its reliability at least under these conditions. Nevertheless, the model is capable to dig in specifically in all the main irreversibility sources. This is the objective of the next section.

## 3. Results

### 3.1. Physically Acceptable Regions

This subsection presents an analysis of the links and constraints among the different variables of the overall system and overall charge/discharge cycle when all the losses that the model considers are taken into account together. It is a key point in a complex thermodynamic system like the one considered as to which are the physically acceptable regions and which are the constraints for the main parameters in order to manage an adequate design.

In each subsystem (heat pump or engine) there are six irreversibility parameters (pressure losses, irreversibilities in the heat transfer from the working fluid to the reservoirs, and losses in expanders/turbines or compressors) and one operation variable, $a_{c}^{HE}$. Moreover, due to the different options for possible storage media, it will be of interest to study the influence of the maximum temperature at which the storage device can operate. This adds up $T_{H}$ as a variable parameter. As it has been addressed in the context of energy storage in liquid media [12] there are physical constraints that define operation regions, that is, the possible values that the operation variables can acquire are restricted and the physical region lastly depends on the values of the irreversibility parameters. The combination of these values might prevent the system from reaching certain operation regimes. So far, the joint variation of such parameters has been analyzed in a multiobjective optimization scheme, obtaining a Pareto front for a given irreversibilities configuration [13]. Here, the analysis will be focused on the influence of each parameter in a certain operation regime, where the operation regime refers to the maximum available value of an objective function and not the global maximum, which might not be available.

Table 1 summarizes the constraints among the temperatures of the working fluid and pressure ratios that must be satisfied to obtain physically acceptable values of the heat transfers and thermodynamic figures of merit (COP values, heat engine efficiency, and round-trip efficiency).

**Figure 6:**
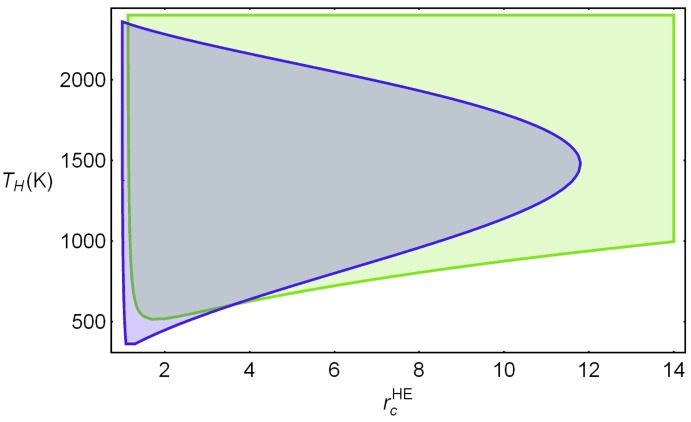
Physically acceptable regions for the two parameters chosen as independent variables, $r_{c}^{HE}$ and $T_{H}$. Parameters quantifying irreversibilities at a representative design point are: [21,22,23]: $\epsilon_{c}^{HP,\;HE}=\epsilon_{t}^{HP,\;HE}=0.95$, $\epsilon_{H}^{HP,\;HE}=\epsilon_{L}^{HP,\;HE}=0.95$, and $\rho_{H}^{HP,\;HE}=\rho_{L}^{HP,\;HE}=0.98$. Green region: 0≤Φ≤1. Blue: Combinations of $T_{H}$ and $r_{c}^{HE}$ satisfying the temperature bounds in Table 1. The intersection between both represents the physically allowable region.

Figure 6 shows the acceptable region considering the specific constraints and for a representative combination of parameters. The intersection is the physically adequate region to perform optimization and sensitivity analyses. The set of parameters characterizing irreversibilities range from typically (and optimistic) values in the literature and are considered for the so-called reference design point in the subsequent analyses [21,22,23]. They are given by: $\epsilon_{c}^{HP,\;HE}=\epsilon_{t}^{HP,\;HE}=0.95$, $\epsilon_{H}^{HP,\;HE}=\epsilon_{L}^{HP,\;HE}=0.95$, and $\rho_{H}^{HP,\;HE}=\rho_{L}^{HP,\;HE}=0.98$. Further on, a sensitivity analysis showing the importance of these parameters will be shown. In the figure, it was taken $T_{L}=300$ K and the working fluid was considered as monoatomic (for instance Ar) in which refers to its adiabatic coefficient. In other figures, $a_{c}^{HE}$ will be taken as the independent variable. This avoids the election of a particular numerical value for the adiabatic coefficient. The figure shows the allowable correlations between $T_{H}$ and $r_{c}^{HE}$ for the specified set of realistic values of irreversibility parameters. Numerical intervals for both $T_{H}$ and $r_{c}^{HE}$ are realistic and acceptable. Other combinations of irreversibility parameters will lead to slightly different acceptable regions. This will be commented on in detail afterward. Once the possible physical regions are delimited, it is possible to analyze meaningful functions such as round-trip efficiency, net power output, or efficiency.

**Figure 7:**
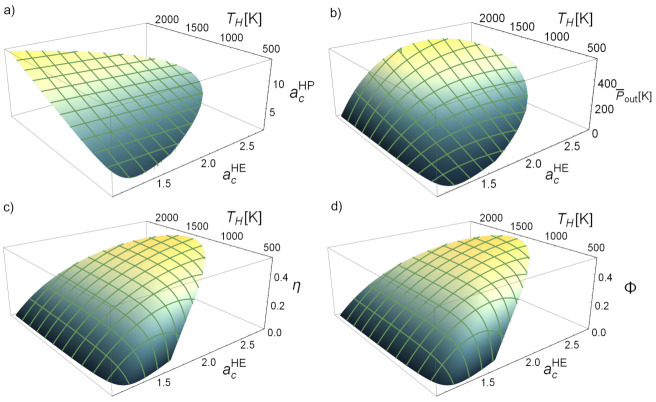
Evolution with the heat engine compressor temperature ratio, $a_{c}^{HE}$, and the equilibrium temperature of the hot storage, $T_{H}$, of some key functions: (**a**) heat pump temperature ratio, (**b**) heat engine power output, ${\bar{P}}_{out}\equiv P_{out}/C_{w}$, (**c**) heat engine efficiency, η, and (**d**) round-trip efficiency, Φ. Irreversibilities parameters are like in Figure 6 and $T_{L}=300$ K.

Figure 7 shows the possible attainable values that can be obtained from the physical region for the power output, HE efficiency, and round-trip efficiency, and also for the compressor temperature ratio of the heat pump. The latter linearly increases with $T_{H}$ as shown in Figure 7a. The net power output obtained in the discharge, ${\bar{P}}_{out}$, (see Figure 7b) increases with $T_{H}$ and has a parabolic behavior with $a_{c}^{HE}$, i.e., there is an optimum value of $a_{c}^{HE}$ maximizing ${\bar{P}}_{out}$ for each value of the temperature of the hot reservoir. The round-trip efficiency (Φ) and the discharge engine efficiency (η) have non-trivial behaviors with the temperature $T_{H}$ and with $a_{c}^{HE}$ (see Figure 7c,d). In general, increasing the temperature $T_{H}$ (with constant $a_{c}^{HE}$) implies increasing these functions, but after a particular temperature the physically acceptable region is narrower and both efficiencies start to decrease. On the other hand, if $T_{H}$ is set constant and $a_{c}^{HE}$ changes, the functions will present a maximum value for a specific $a_{c}^{HE}$. So, optimizing the parameter $a_{c}^{HE}$ and raising the temperature $T_{H}$ seems to be the most beneficial option for not particularly high temperatures as can also be seen in Figure 6, where an increase of $T_{H}$ reduces the acceptable interval for $a_{c}^{HE}$, which is an important consideration to account for. The optimization of these objective functions will be analyzed in the next section.

The thermodynamic functions of interest generally exhibit non-trivial behaviors in the acceptable physical region defined by the considered independent variables and, thus, at least two detailed analyses are in order: an optimization survey for the chosen independent variables for a particular set of irreversibility parameters and a sensitivity analysis of the optimum values of the objective functions with respect to irreversibilities. These studies will be shown in the next subsections.

### 3.2. Optimization of Overall Performance

The influence of irreversibilities in the coupled cycle will be performed in the so-called design point (see the caption of Figure 6), a representative configuration of irreversibilities that serves as a reference to compare the influence of each parameter. The value for the temperature $T_{H}$ is chosen such that $a_{c}^{HE}$ has a large action region, allowing it to perform at several operation regimes and, of course, it should be a realistic temperature for solid heat reservoirs. A value $T_{H}=1000$ K fulfills these requirements.

**Figure 8:**
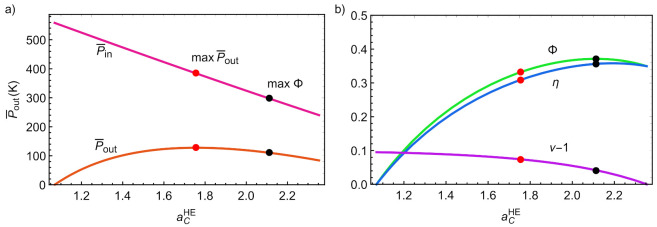
(**a**) Input and output power versus discharge temperature ratio. (**b**) Round-trip efficiency, COP and discharge efficiency versus discharge temperature ratio. Black dots indicate the value of $a_{c}^{HE}$ leading to maximum round-trip efficiency, the red dots those of maximum power output.

Figure 8a represents the input/output powers as a function of $a_{c}^{HE}$. Notice that $P_{in}$ is a monotonic decreasing function whereas $P_{out}$ is parabolic and displays a maximum slightly below $a_{c}^{HE}=1.8$. For a monoatomic working fluid as Ar, it corresponds to a pressure ratio $r_{c}^{HE}≃4.3$ that is a realistic value for actual compressors. For air, that value of $r_{c}^{HE}$ would be about 7.8, which is also an acceptable value. Figure 8b depicts the round-trip efficiency, the discharge cycle efficiency (η), and the charge cycle coefficient of performance (COP) (minus unity, to scale the graphic and compare the functions). The black point in all curves in Figure 8 corresponds to the maximum round-trip efficiency and the red point corresponds to maximum $P_{out}$ ($max\;P_{out}$). As it can be seen, unlike in the endoreversible limit, both maxima are reached at different $a_{c}^{HE}$ values, which nonetheless is in agreement with previously analyzed PTES systems [12,13]. It is interesting to note that (see Figure 8b) the maximum round trip leads to a high value of η, not exactly the corresponding maximum, but a numerical value close to it. Numerically, the maximum round-trip is obtained at a pressure ratio $r_{c}^{HE}≃6.4$ for monoatomic gases and $r_{c}^{HE}≃13.4$ for diatomic ones, values that are above those leading to maximum power output.

**Figure 9:**
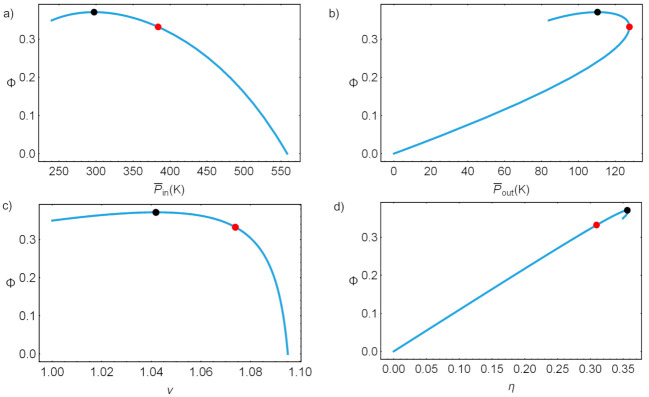
Parametric loops: in (**a**) round-trip efficiency versus ${\bar{P}}_{in}$, in (**b**) round-trip efficiency versus ${\bar{P}}_{out}$, in (**c**) round-trip efficiency versus the HP COP and in (**d**) round-trip efficiency versus the discharge efficiency. Black dots indicate the value of $a_{c}^{HE}$ leading to maximum round-trip efficiency, and red dots those of maximum power output.

Figure 9 shows the parametric plots of round-trip versus several other thermodynamic functions ranging on all possible $a_{c}^{HE}$ values. Maximum ${\bar{P}}_{out}$ and maxΦ are indicated by black and red dots, the region in between is usually considered as the optimal design region of the device. As it will be seen later, the physical constraints might forbid the system from reaching such operational states.

### 3.3. Irreversibility Analysis

For Brayton-like HP and HE cycles, optimistic values for the irreversibilities can be considered in the ranges depicted in Table 2. The sensitivity of the main operation regimes to different parameters and for charge/discharge stages has not yet been carefully addressed and, thus, it is the objective of this subsection.

**Figure 10:**
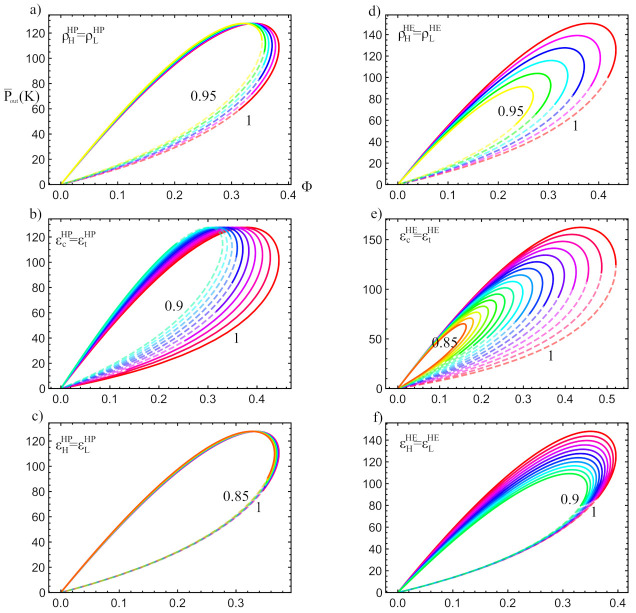
Parametric plots of ${\bar{P}}_{out}$ vs Φ over all available $a_{c}^{HE}$ values ($a_{c}^{HE}$ increases clockwise). Solid lines indicate physically acceptable regimes meanwhile dashed curves denote the not available operation states. In each plot the corresponding irreversibility parameter varies taking the values shown in Table 2: (**a**) $\rho_{H,L}^{HP}$, (**b**) $\epsilon_{c,t}^{HP}$, (**c**) $\epsilon_{H,L}^{HP}$, (**d**) $\rho_{H,L}^{HE}$, (**e**) $\epsilon_{c,t}^{HE}$, (**f**) $\epsilon_{H,L}^{HE}$. Notice the limit of the physical region when the dashed and semi-transparent lines begin. In all figures $T_{H}=1000$ K and $T_{L}=300$ K.

The effects on the power output $P_{out}$ and in the round-trip (Φ) of the coupled cycle stemming from these parameters is presented in Figure 10. In Figure 10a–c the influence of the pressure drops is shown, as are the expander and compressor efficiencies, and the effectiveness of heat transfers, respectively, over the charge phase. Similarly, in Figure 10d–f it is shown the influence of the pressure drops; turbine and compressor efficiencies, and the effectiveness, respectively, over the discharge phase. In each figure, solid curves represent the power and round-trip values in the physically acceptable region. Each curve starts at $P_{out}=0$ with the minimum value of $a_{c}^{HE}$. As $a_{c}^{HE}$ increases, the curve goes through the parametric loop in a clockwise direction. At a certain point, a maximum value of $a_{c}^{HE}$ is reached (in the sense of Figure 6), which leads to the end of the physical region of our cycle (dashed lines begins) and the mentioned physical and practical constraints are not fulfilled. In other words, for each combination of irreversibility parameters, an intersection area as that shown in Figure 6 is built. Dashed lines in the figures correspond to points outside that region, i.e., are not acceptable.

As it can be seen in a general way in Figure 10, the overall cycle is more sensitive to irreversibilities in the discharge cycle than those of the charge (left versus right column). The irreversibilities in the components of the charge cycle (compressor, expander, and heat exchangers) do not directly affect the thermodynamic functions of the discharge cycle, such as the maximum power output. However, they produce a large reduction in the acceptable physical region, especially for large irreversibilities. This reduction in the acceptable physical region is shown as an increase in the length of the dashed lines and a reduction in the length of the solid lines. When the reduction of the acceptable physical region becomes very noticeable, the values of the parameter $a_{c}^{HE}$ which maximize the power output and the HE efficiency might no longer be included in the physical region, decreasing the maximum available $P_{out}$ and Φ. Therefore, indirectly and through the decrease in the acceptable physical region, the irreversibilities of the HP affect the discharge thermodynamic functions. In Figure 10b it is observed how $\epsilon_{c,t}^{HP}$ reduce the acceptable physical region more abruptly than the external irreversibilities, $\epsilon_{H,L}^{HP}$, shown in Figure 10c. This same behavior is reproduced for the HE in the 2nd column.

### 3.4. Sensitivity Analysis

**Figure 11:**
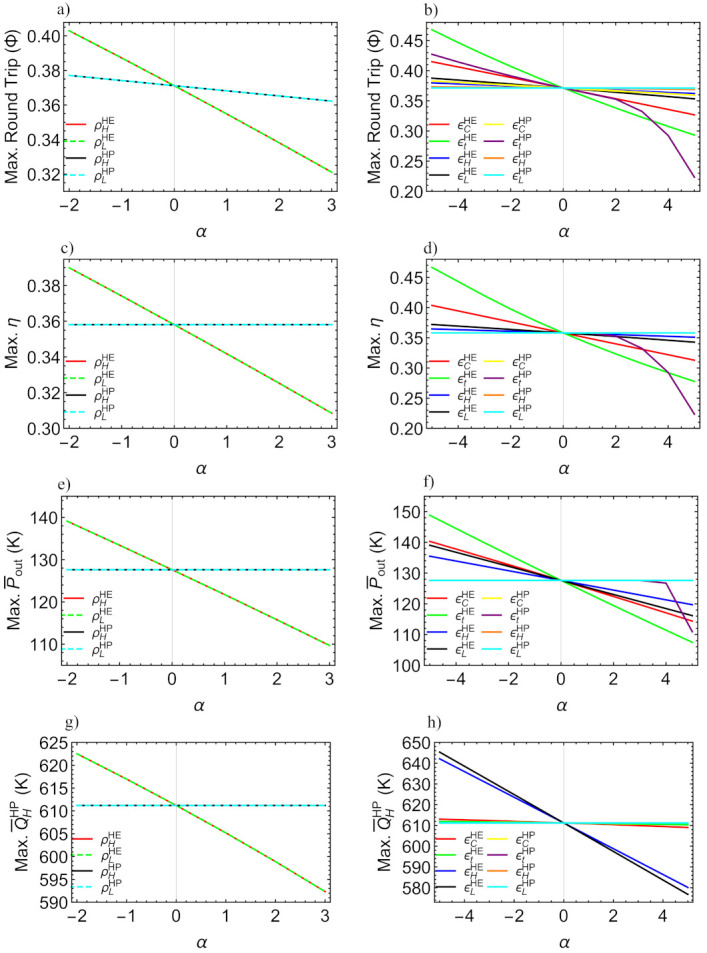
Sensitivity analysis of the maximum round-trip efficiency (**a**,**b**), HE efficiency (**c**,**d**), power output (**e**,**f**) and pumped heat (**g**,**h**) with respect to the reference/design irreversible configuration. The parameter $\alpha \equiv \left(Irr^{*}-Irr\right)\times 100$ measures the relative changes of each parameter with respect to the design point. See text for details.

In this section an analysis of the sensitivity of the maximum round-trip, discharge performance, power output and pumped heat with respect to losses will be performed. This will provide a landscape for irreversibilities relevance/influence for each figure of merit. For this, a reference configuration will be considered (design point shown in caption of Figure 6 and $T_{H}=1000$ K). In Figure 11 such effects on the maxima are depicted. The parameter $\alpha \equiv \left(Irr^{*}-Irr\right)\times 100$ is introduced as a measure of the irreversibilities variation with respect to the design point. Irr refers to each parameter and $Irr^{*}$ is the value at the reference/design point. In this way, at the design point: α=0; and according Table 2 all ϵ coefficients vary from 1 (α=−5) to 0.9 (α=+5). For the pressure drops (ρ) will be varied from 1 (α=−2) to 0.95 (α=+3). Figure 11a shows the maximum round-trip efficiency when pressure drops vary from ideal conditions, down to 0.95. Figure 11b shows the effects on the maximum round-trip efficiency due to the increment or decrement of all the efficiencies. In Figure 11c the maximum efficiency is depicted for variation of pressure drops and in Figure 11d the same is done for variations of each ϵ. This sensitivity analysis is done also for the maximum power density, ${\bar{P}}_{out}$, in Figure 11e for variation in the ρ’s and in Figure 11f for variations in all ϵ’s. Finally, the sensitivity of the maximum pumped heat is depicted in Figure 11g for variations of each ρ and in Figure 11h for variations in each ϵ.

It was found that the irreversibilities in the expander/turbine, $\epsilon_{t}$, are the most determinant in the drop of the maximum values for Φ, η and $P_{out}$ (see Figure 11b,d,f). Especially the losses in the expander of the charge cycle, $\epsilon_{t}^{HP}$, are more determinant than those of the turbine in the discharge, $\epsilon_{t}^{HE}$ whose influence stem from the operation region reduction. It is also noticeable that the reduction in the maximum values of those functions with $\epsilon_{t}^{HP}$ over design point values is clearly not linear (see purple curves). From the Figure 11a,b, it can be seen that by reducing the irreversibilities with respect to the considered design point, maximum round-trip efficiencies over 0.4 could be obtained, which is a good result compared to previously reported values.

Notice that the maximum heat pumped strongly depends on the external irreversibilities of the discharge cycle, $\epsilon_{H}^{HE}$ and $\epsilon_{L}^{HE}$ (see black and blue curves in Figure 11h). Meanwhile, the influence of $\epsilon_{H}^{HP}$ is small, and as expected, there is no influence from $\epsilon_{L}^{HP}$. The relevance of the HE in the pumped heat is due to the linking between both subsystems, stemming from the constraint $Q_{H}^{HP}=Q_{H}^{HE}$. This behavior is reproduced in the case of pressure drops; those of the HP do not influence the maximum $Q_{H}$, but the influence of the HE is significant.

## 4. Discussion and Conclusions

In the present work, a PTES system based on a coupled Brayton-like HE and HP has been analyzed. This includes the analysis of 12 parameters associated with irreversibilities, eight for internal irreversibilities (pressure losses and expanders/turbines and compressors losses), and four for external ones (heat transfers with the reservoirs). One variable, $a_{c}^{HE}$, the pressure ratio of the compressors in the discharge cycle, is used as an independent variable to establish the operation regime. Meanwhile, $a_{c}^{HP}$ is given by the constraint $Q_{H}^{H}P=Q_{H}^{HE}$.

Unlike in the case of energy storage in liquid media with variable temperature in the TES [13], here it is shown that in the present model it is not possible to simultaneously optimize power output and round-trip efficiency (see Figure 8 and Figure 9), mostly because the physically acceptable region is wider. In the case reported in [13], the physical region was strongly constrained by the thermal properties of the molten salts (lower and upper limits for the temperatures of the reservoirs). Here the absence of this constraint allows for a wider range of operation regimes in the Φ-$P_{out}$ curve. Additionally, increasing the temperature of the hot storage ($T_{H}$) allows for better results in *P*, η, and Φ altogether. However, above a certain value, the increase of $T_{H}$ reduces the acceptable physical region, restraining the range of acceptable values for $a_{c}^{HE}$.

The internal irreversibilities, when compared with the external ones, show the following remarkable double effect:

- The values of the internal irreversibilities considerably reduce the maximum achievable power output and round-trip efficiency with respect to the same values for the external irreversibilities.
- The internal irreversibilities greatly reduce the range of acceptable values for $a_{c}^{HE}$; affecting the available operation regimes. In this way, the irreversibilities of the discharge can influence significantly the thermodynamic functions of the charge by severely reducing its acceptable physical region.

Some points can also be remarked from the performed sensitivity analysis:

- The expander/turbine is the most determinant element, especially the expander of the charge cycle is more decisive than the turbine of the discharge cycle.
- Not all irreversibilities exhibit an effect on all thermodynamic functions. The irreversibilities of the charge cycle do not influence the functions of the discharge other than in the case when they considerably reduce the physical region.
- By reducing irreversibilities it is possible to reach maximum values of the round-trip efficiency around 40% or even above. This is a significant result that should be considered for further studies on this type of model.

By establishing the most relevant components affecting the main energetic functions, this analysis might be used to focus on specific improvements in the turbomachinery. The cost of improving specific components might be weighted by its energetic relevance.

Future perspectives include analyzing the constraints imposed by the external sources (wind, photovoltaic, or others) in the inlet power in the charge phase, studying in detail the operation strategies considering realistic storage times, and including a more detailed analysis of the peculiarities of storage reservoirs and transient regimes.

## Figures and Tables

**Table 1:** Bounds to be considered in the coupled charge-discharge system.

Overall Cycle Constraints and Bounds	
Heat conservation	$Q_{H}^{HP}=Q_{H}^{HE}\;\Rightarrow \;T_{2}^{HP}-T_{1}^{HP}=T_{2}^{HE}-T_{1}^{HE}$
Charge conditions	$0\leq T_{4}^{HP}\leq T_{3}^{HP}\leq T_{L}\leq T_{H}\leq T_{1}^{HP}\leq T_{2}^{HP}$
Discharge conditions for $T_{3}^{HE}$	$T_{L}\leq T_{4}^{HE}\leq T_{3}^{HE}\leq T_{2}^{HE}\leq T_{H}$
Discharge conditions for $T_{1}^{HE}$	$T_{L}\leq T_{4}^{HE}\leq T_{1}^{HE}\leq T_{2}^{HE}\leq T_{H}$
Energy conservation	$0\leq \Phi \leq 1\;\Rightarrow \;0\leq \frac{T_{2}^{HE}-T_{1}^{HE}-T_{3}^{HE}+T_{4}^{HE}}{T_{2}^{HP}-T_{1}^{HP}-T_{3}^{HP}+T_{4}^{HP}}\leq 1$
Carnot maximum efficiency	$0\leq \eta \leq 1-T_{L}/T_{H}\;$ & $\;1\leq \nu \leq {\left(1-T_{L}/T_{H}\right)}^{-1}$
Pressure and temperature ratios links	$a_{t}^{HP}=a_{c}^{HP}\rho_{H}^{HP}\rho_{L}^{HP}$
Pressure and temperature ratios links	$a_{t}^{HE}=a_{c}^{HE}\rho_{H}^{HE}\rho_{L}^{HE}$

**Table 2:** Typical values of irreversibilities.

Irreversibilities	Interval
$\epsilon_{c,t}^{HP,\;HE}$	$\in \left[0.85,1\right]$
$\epsilon_{H,L}^{HP,\;HE}$	$\in \left[0.85,1\right]$
$\rho_{H,L}^{HP,\;HE}$	$\in \left[0.9,1\right]$

## Data Availability

Not applicable.

## Abbreviations

**Acronyms**	
COP	Coefficient of performance
HE	Heat engine
HP	Heat pump
PTES	Pumped Thermal Energy Storage
RTE	Round-trip efficiency
TES	Thermal Energy Storage
**Symbols**	
*a*	Temperature ratio
*C*	Heat capacity (J/Ks)
$\dot{Q}$	Heat flow (J/s)
*W*	Work (J)
*P*	Power (J/s)
${\bar{P}}_{out}$	Specific Power $P/C_{w}$ (K)
κ	Dimensionless factor $\left(\frac{\gamma -1}{\gamma }\right)$
$\dot{m}$	Mass flow (kg/s)
*p*	Pressure (atm)
*r*	Pressure ratio
*T*	Temperature (K)
**Greek Letters**	
γ	Adiabatic coefficient of ideal gas
ϵ	Efficiency parameter
η	Discharge efficiency
ν	Coefficient of performance
ρ	Pressure drops coefficient
Φ	Round-trip efficiency
**Subscript**	
*w*	Working fluid
*c*	Compressor
*H*	Hot storage
*L*	Cold storage
*t*	Expander/turbine

## References

[1] Benato A., Stoppato A. (2018). Pumped Thermal Electricity Storage: A technology overview. Therm. Sci. Eng. Prog..

[2] Benato A. (2017). Performance and cost evaluation of an innovative Pumped Thermal Electricity Storage power system. Energy.

[3] Gallo A.B., Simões Moreira J.R., Costa H.K.M., Santos M.M., Moutinho dos Santos E. (2016). Energy Storage in the energy transition context: A technology review. Renew. Sust. Energy Rev..

[4] Guney M.S., Tepe Y. (2017). Classification and assessment of energy storage systems. Renew. Sust. Energy Rev..

[5] Dumont O., Frate G.F., Pillai A., Lecompte S., De paepe M., Lemort V. (2020). Carnot battery technology: A state-of-the-art review. J. Energy Storage.

[6] Cabeza L.F., Solé A., Barreneche C. (2016). Review on sorption materials and technologies for heat pums and thermal energy storage. Renew. Energy.

[7] Davenne T.R., Peters B.M. (2020). An Analysis of Pumped Thermal Energy Storage With De-coupled Thermal Stores. Front. Energy Res..

[8] Guo J., Cai L., Chen J., Zhou Y. (2016). Performance evaluation and parametric choice criteria of a Brayton pumped thermal electricity storage system. Energy.

[9] Laughlin R.B. (2017). Pumped thermal grid storage with heat exchange. J. Renew. Sust. Energy Rev..

[10] Esence T., Bruch A., Molina S., Stutz B., Fourmigué J.F. (2017). A review on experience feedback and numerical modeling of packed-bed thermal energy storage systems. Sol. Energy.

[11] McTigue J.D., White A.J., Markides C.N. (2015). Parametric studies and optimisation of pumped thermal electricity storage. Appl. Energy.

[12] Salomone-González D., González-Ayala J., Medina A., Roco J.M.M., Curto-Risso P.L., Hernández A.C. (2020). Pumped heat energy storage with liquid media: Thermodynamic assessment by a Brayton-like model. Energy Conv. Manag..

[13] González-Ayala J., Salomone-González D., Medina A., Roco J.M.M., Curto-Risso P.L., Hernández A.C. (2021). Multicriteria optimization of Brayton-like pumped thermal electricity storage with liquid media. J. Energy Storage.

[14] Wang L., Lin X., Chai L., Peng L., Yu D., Chen H. (2019). Cyclic transient behavior of the Joule-Brayton based pumped heat electricity storage: Modeling and analysis. Renew. Sust. Energy Rev..

[15] Zhang Y., Wang Z. (2021). Comparative study on optimized round-trip efficiency of pumped thermaland pumped cryogenic electricity storages. Energy Conv. Manag..

[16] Thess A. (2013). Thermodynamic Efficiency of Pumped Heat Electricity Storage. Phys. Rev. Lett..

[17] Chen J., Guo J. (2016). Comment on “Thermodynamic Efficiency of PumpedHeat Electricity Storage”. Phys. Rev. Lett..

[18] Gonzalez-Ayala J., Arias-Hernandez L.A., Angulo-Brown F. (2013). Connection between maximum-work and maximum-power thermal cycles. Phys. Rev. E.

[19] Gonzalez-Ayala J., Roco J.M.M., Medina A., Hernández A.C. (2017). Carnot-Like Heat Engines Versus Low-Dissipation Models. Entropy.

[20] Gonzalez-Ayala J., Roco J.M.M., Hernández A.C. (2018). Entropy generation and unified optimization of Carnot-like and low-dissipation refrigerators. Phys. Rev. E.

[21] Roco J.M.M., Velasco S., Medina A., Hernández A.C. (1997). Optimum performance of a regenerative Brayton thermal cycle. J. Appl. Phys..

[22] Sánchez-Orgaz S., Medina A., Hernández A.C. (2010). Thermodynamic model and optimization of a multi-step irreversible Brayton cycle. Energy Conv. Manag..

[23] Ahmadi M.H., Ahmadi M.A., Pourfayaz F., Bidi M. (2016). Thermodynamic analysis and optimization for an irreversible heat pump working on reversed Brayton cycle. Energy Conv. Manag..

